# L-Leucine Promotes STAT1 and ISGs Expression in TGEV-Infected IPEC-J2 Cells *via* mTOR Activation

**DOI:** 10.3389/fimmu.2021.656573

**Published:** 2021-07-22

**Authors:** Jian Du, Daiwen Chen, Bing Yu, Jun He, Jie Yu, Xiangbing Mao, Yuheng Luo, Ping Zheng, Junqiu Luo

**Affiliations:** Key Laboratory for Animal Disease-Resistance Nutrition, Animal Nutrition Institute, Sichuan Agricultural University, Chengdu, China

**Keywords:** L-leucine, transmissible gastroenteritis virus, mammalian target of rapamycin, signal transducer and activator of transcription 1, IPEC-J2 cells

## Abstract

L-leucine (Leu), as one of the effective amino acids to activate the mTOR signaling pathway, can alleviate transmissible gastroenteritis virus (TGEV) infection. However, the underlying mechanism by which Leu alleviates the virus infection has not been fully characterized. In particular, how Leu impacts TGEV replication through mTOR signaling has yet to be elucidated. In the present study, we found that TGEV proliferated efficiently in intestinal porcine epithelial cells (IPEC-J2 cells) as evidenced by the increase in viral contents by flow cytometry, the inhibition of cell proliferation by CCK-8 assay as well as the reduction of PCNA level by western blot. Besides, western blot analysis showed that STAT1 expression was markedly reduced in TGEV-infected cells. The results of ELISA revealed the inhibition of ISGs (ISG56, MxA, and PKR) expressions by TGEV infection. TGEV-induced mTOR and its downstream p70 S6K and 4E-BP1, STAT1 and ISGs downregulation were blocked by an mTOR activator-MHY1485 but not by an mTOR inhibitor-RAPA. Concurrently, mTOR activation by MHY1485 reduced the contents of TGEV and vice versa. Furthermore, Leu reversed the inhibition of STAT1 and ISGs by activating mTOR and its downstream p70 S6K and 4E-BP1 in TEGV-infected cells. Our findings demonstrated that Leu promoted the expressions of STAT1 and ISGs *via* activating mTOR signaling in IPEC-J2 cells, aiming to prevent TGEV infection.

## Introduction

Transmissible gastroenteritis virus (TGEV), which belongs to the genus *Alphacoronavirus*, is an enveloped, single-stranded, positive-sense RNA virus (1, 2). TGEV replicates in the cytoplasm of differentiated enterocytes covering the small intestinal villi and causes acute enteritis in swine of all ages (3, 4). The common clinical manifestations are anorexia, vomiting, watery diarrhea, dehydration, and weight loss in piglets. Specifically, the mortality rate of seropositive suckling piglets may reach up to 100% during epidemics (5). Despite the availability of vaccines, outbreaks can be encountered globally and cause great economic losses in the swine industry (6).

In the process of viral infection and replication, innate immunity, as the first line of defense, detects pathogen-associated molecular patterns (PAMPs) of invading viruses by different pattern recognition receptors (PRRs) and responds accordingly by producing a series of effector molecules or inflammatory elements against viral invasion (7, 8). Among most innate immune responses, IFN-I signaling is one of the most important antiviral innate immunity to antagonize virus infection. Phosphorylation of STAT1 on tyrosine 701 plays a pivotal role in activating the IFN-I signaling pathway (9–11). Even though IFNs exert antiviral activity during viral infection, the ability to suppress innate immune responses provides invading viruses with the opportunity to replicate and establish a productive infection (12–14). A previous study revealed that porcine epidemic diarrhea virus (PEDV), which is genetically related to TGEV, can inhibit STAT1 expression through the ubiquitin-proteasome targeting degradation system (15).

A great deal of evidence supports the notion of mTOR function in maintaining the structure of the intestinal mucosa and in the self-renewal of stem cells. Emerging studies have begun to shed light on the interplay between mTOR signaling and innate immune signaling transductions, arguing for their possible roles in the regulation of host antiviral responses (16, 17). Several studies have indicated that mTOR could regulate STAT1 expression (18–20). However, how mTOR regulation influences STAT1 expression in TGEV-infected cells remains unknown.

In the meantime, identifying an effective treatment for TGEV pathogens is of utmost importance in terms of the antibiotic prohibition. Because the possibility of viral gene mutation, general therapeutic drawbacks of vaccines, and effects of prescription drugs only on clinical symptoms still put the host at risk, the development of effective nutritional regimens targeting mTOR/STAT1 activation has become one of the integral means to antagonize TGEV infection (21). Recent studies on pigs have shown that branched-chain amino acids (BCAAs) improve intestinal integrity and function and modify the production of immunoregulatory cytokines to protect the host from different diseases (22, 23). Among the three BCAAs, L-leucine (Leu) earns the greatest reputation for its unique function of activating the mTOR signaling pathway (24, 25). In the present study, we investigated the role of mTOR in regulating the IFN-I response. Our results clearly demonstrated that TGEV inhibited the expressions of mTOR, p70 S6K, 4E-BP1, STAT1 and ISGs. Mechanistically, mTOR activity regulation both by its own activator and Leu could alleviate TGEV infection *via* increasing in STAT1 and ISGs expression. On the contrary, the specific inhibitor of mTOR promoted TGEV replication by reducing STAT1 and ISGs expressions.

## Materials and Methods

### Cells and Virus

TGEV strain WH-1 (GenBank accession no. HQ462571.1) was kindly offered from the College of Veterinary Medicine, Sichuan Agricultural University (26). Virus titers were determined by 50% tissue culture infective doses (TCID_50_) assay. Intestinal porcine epithelial cells (IPEC-J2 cells) were cultured and maintained in Dulbecco’s modified Eagle’s F12 Ham medium (DMEM-F12; Gibco, USA) supplemented with 10% heat-inactivated fetal bovine serum (FBS; Gibco, USA) and 1% penicillin–streptomycin (Gibco, USA) at 37°C in a humidified 5% CO_2_ incubator. The swine testis cells (ST-0746) were cultured and maintained in DMEM (Gibco, USA) and used to amplify TGEV.

### Reagents and Antibodies

Polyclonal antibodies against STAT1, phospho-STAT1 (Tyr701) Rabbit mAb, PCNA antibody Mouse mAb, and the β-actin antibody were purchased from Cell Signaling Technology, USA. Recombinant human IFN-β was purchased from Peprotech, USA. Rapamycin (RAPA) and MHY1485 were purchased from Selleck Chemicals, USA. Leu was purchased from Sigma-Aldrich, USA. Before TGEV infection, IPEC-J2 cells were pretreated with 10 nM RAPA or 10 μM MHY1485 for 30 min. Differently, the cells were starved for 3 h in the Earle’s Balanced Salt Solution (EBSS; Gibco, USA) and then pretreated with 10 mM Leu before TGEV infection. TGE Virus Antibody (1-Q-17) was purchased from Santa Cruz Biotechnology, USA. Goat Anti-Mouse IgG H&L (Alexa Fluor^®^ 488) was purchased from Abcam, USA.

### CCK-8 Assay

IPEC-J2 cells were seeded on glass coverslips (Corning, USA) in 96-well plates (1 × 10^4^ cells per well). After reaching 80% confluence, the medium was replaced with IPEC-J2 cell starvation medium, followed by infection with TGEV (MOI = 5) for 1 h. Then, the virus culture medium was replaced by IPEC-J2 cell starvation medium. The cells were cultured for 0, 6, 12, 24, 36, and 48 h, respectively. After that, 10 μl of CCK-8 solution was added into each well and then cultured in the cell incubator for 2 h. The absorbance at 450 nm was determined by enzyme-linked immunosorbent assay.

### The Kinetic Curve of TGEV Replication in IPEC-J2 Cells

ST cells were infected with TGEV (MOI = 5) at different time points (6, 12, 24, 36, 48 h). The virus was harvested at different time points by repeated freezing and thawing for 3 times, followed by centrifugation at 3,000 r/min for 10 min. Then, the supernatant was filtered with a 0.22 μm filter to preserve at −80°C. The virus titers were measured in IPEC-J2 cells. TCID_50_ was calculated by the Reed–Muench method to draw the kinetic curve of TGEV replication.

### Flow Cytometry Analysis

IPEC-J2 cells were collected in the EP tubes with 1 ml PBS and centrifuged for 5 min at 4°C in 350 relative centrifugal force (rcf). Then we removed the supernatant and resuspended and fixed cells with 4% paraformaldehyde for 15 min. After 500 rcf centrifugation at 4°C for 5 min, 100 μl TGEV primary antibody (0.05% Titonx-100: primary antibody = 100: 1 v/v) was added. Two hours later at room temperature, cells were centrifuged for 5 min at 4°C in 500 rcf. Next, the supernatant was removed and we added 100 μl second antibody (PBS: second antibody = 1,000:1) in the dark condition. After storage at room temperature for 1 h, we added 900 μl PBS for centrifugation. Then the supernatant was removed and 500 μl PBS was added to resuspend cells for detection.

### RT-qPCR

Total cellular RNA was extracted with TRIzol Reagent (Invitrogen, USA) from TGEV-infected IPEC-J2 cells and an aliquot (1 μg) was reverse-transcribed into cDNA using a PrimeScript™ RT Reagent Kit with gDNA Eraser (TaKaRa, Japan). The obtained cDNA was then used as the template in the SYBR Green I PCR assay (Applied Biosystems, CA). Real-time qPCR was performed for STAT1 and a house-keeping gene (GAPDH) according to standard protocols with the primers indicated in **Table S1**.

### Western Blot Analysis

Briefly, IPEC-J2 cells were collected in the 1.5 ml EP tube for centrifugation at 500 rcf for 5 min at 4°C. After removing the supernatant, cells were mixed with RIPA lysis buffer (Beyotime, China) containing PMSF (Sigma-Aldrich, US) and kept on the ice for 30 min. Ultrasonication was then performed to break cells, followed by centrifugation at 10,000 rcf for 15 min at 4°C. The proteins in the supernatant containing with 4× Laemmli Sample Buffer (Bio-Rad, USA) were denatured in the 98°C-metal bath for 10 min. Equal amounts of samples were then subjected to SDS-PAGE, and the expressions of STAT1 and p-STAT1 protein were examined by western blot analysis using the indicated antibodies. The expression of β-actin was detected to verify equal protein sample loading.

### Enzyme-Linked Immunosorbent Assay (ELISA)

The supernatants of different treatments were collected to determine the concentrations of ISGs (ISG15, ISG56, MxA and PKR) using spectrophotometric kits in line with the manufacturer’s instructions (MEIMIAN, Jiangsu, China). The protein concentrations were expressed pg/ml or ng/ml.

### Statistical Analysis

All data were expressed as means ± standard error of means (SEMs). The statistical significance was tested by unpaired two-tailed Student’s *T* test and/or one-way analysis of variance (ANOVA) using IBM SPSS Statistics version 20.0 (IBM, USA). When there was a significant interaction, *post hoc* testing was conducted using Tukey’s multiple comparison test. A *p*-value of <0.05 was considered statistically significant.

## Results

### IPEC-J2 Cells Are Susceptible to TGEV

**Figure 1:**
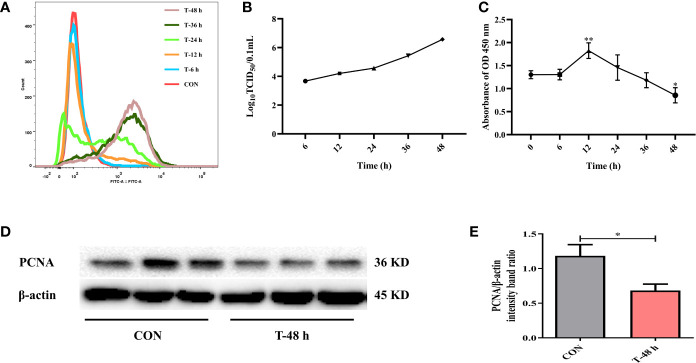
TGEV infection in IPEC-J2 cells. **(A)** The contents of TGEV at 0, 6, 12, 24, 36, and 48 hpi were analyzed *via* flow cytometry. **(B)** The viral titers of TGEV infected IPEC-J2 cells at 6, 12, 24, 36, and 48 hpi. **(C)** CCK-8 assay was used to detect the cell proliferation after TGEV infection at 6, 12, 24, 36, and 48 hpi. Data express the mean ± SEM (n = 3). **(D)** The PCNA level was detected by western blot. **(E)** Quantitation of bands to demonstrate the protein level of PCNA. Data express the mean ± SEM (n = 3). The symbol * indicates statistically significant differences (*P <* 0.05), and the symbol ** indicates statistically very significant differences (*P* < 0.01).

Firstly, to determine whether TGEV could proliferate in IPEC-J2 cells, cells were incubated with TGEV (MOI = 5) for 0, 6, 12, 24, 36, and 48 h. We observed a massive viral replication at 24 h post-infection (hpi). Moreover, the contents of TGEV increased in a time-dependent manner (**Figure 1A**). Then, the percentage of TGEV infected IPEC-J2 cells was examined. At first, virus particles were assembled and released into the extracellular matrix; however, the titer of TGEV was very low. During 24 to 48 h, the virus replicated rapidly. In particular, at 48 hpi, the TCID_50_ of virus reached the highest level (10^−6.57^/100 μl), which is close to the TCID_50_ (10^−6.8^/100 μl) measured by TGEV in ST cells (**Figure 1B**).

Next, CCK-8 assays were performed to assess cell proliferation at different time points after TGEV infection (MOI = 5). The results revealed that cell proliferation was boosted at 12 hpi, but was remarkably suppressed afterward (**Figure 1C**). Besides, the protein expression of PCNA was dramatically inhibited by TGEV infection at 48 hpi (**Figures 1D, E**).

### TGEV Replication Counteracts IFN-I Signaling Pathway

**Figure 2:**
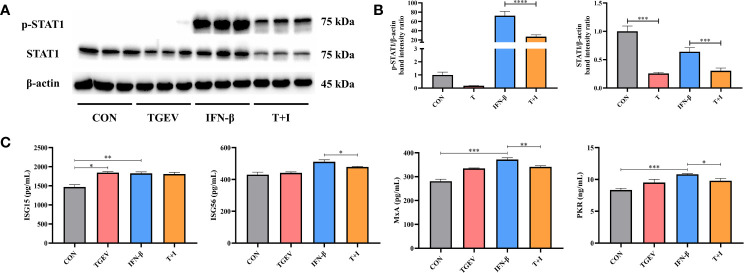
IFN-I signaling pathway was blocked by TGEV infection. **(A)** Both p-STAT1 and T-STAT1 levels were detected by western blot. **(B)** Quantitation of bands to demonstrate the protein level of p-STAT1 and T-STAT1. Data express the means ± SEMs (n = 3). **(C)** The expressions of ISGs (ISG15, ISG56, MxA and PKR) were analyzed *via* ELISA. Data express the means ± SEMs (n = 3). The symbol * indicates statistically significant differences (*P* < 0.05), **P < 0.01, ***P < 0.001 and ****P < 0.0001.

To assess the effect of TGEV infection on STAT1 activation, the levels of phosphorylated STAT1 (p-STAT1) and STAT1 were examined in TGEV-infected cells. Since the ability of IFN-β to induce STAT1 phosphorylation has been well documented previously, recombinant IFN-β was exogenously administered in the positive control (27). Western blot analysis revealed that compared with the positive control, TGEV significantly decreased the expressions of p-STAT1 and STAT1 (**Figures 2A, B**). Similarly, the concentrations of ISGs (ISG56, MxA, and PKR) related to STAT1 activation were also decreased by TGEV (**Figure 2C**). Taken together, TGEV could inhibit the activation of STAT1 regulated by IFN-β and its downstream ISGs to block the IFN-I signaling pathway.

### mTOR Regulation Is Involved in Control of TGEV Replication

**Figure 3:**
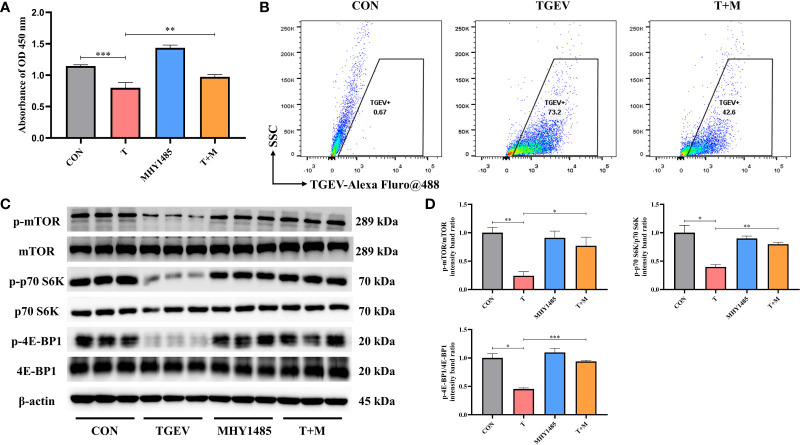
MHY1485 attenuated the TGEV infection by activating mTOR signaling. **(A)** CCK-8 assay was used to detect the proliferation of IPEC-J2 cells pretreated with 10 μM MHY1485, followed by TGEV infection. Data express the mean ± SEM (n = 3). **(B)** The contents of TGEV were analyzed *via* flow cytometry in the IPEC-J2 cells pretreated with 10 μM MHY1485, followed by TGEV infection. **(C)** The protein levels of p-mTOR, mTOR, p-p70 S6K, p70 S6K, p-4E-BP1 and 4E-BP-1 were detected by western blot. **(D)** Quantitation of bands to demonstrate the protein level of p-mTOR/mTOR, p-p70 S6K/p70 S6K and p-4E-BP1. Data express the means ± SEMs (n = 3). The symbol * indicates statistically significant differences (P < 0.05), **P < 0.01 and ***P < 0.001.

**Figure 4:**
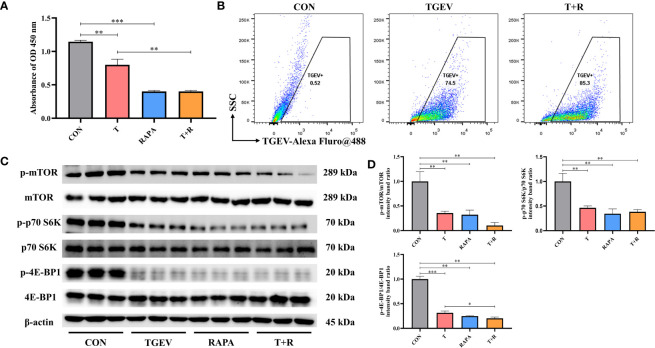
RAPA promoted the TGEV replication by inhibiting mTOR signaling. **(A)** CCK-8 assay was used to detect the proliferation of IPEC-J2 cells pretreated with 10 nM RAPA, followed by TGEV infection. Data express the mean ± SEM (n = 3). **(B)** The contents of TGEV were analyzed *via* flow cytometry in the IPEC-J2 cells pretreated with 10 nM RAPA, followed by TGEV infection. **(C)** The protein levels of p-mTOR, mTOR, p-p70 S6K, p70 S6K, p-4E-BP1 and 4E-BP-1 were detected by western blot. **(D)** Quantitation of bands to demonstrate the protein level of p-mTOR/mTOR, p-p70 S6K/p70 S6K and p-4E-BP1. Data express the means ± SEMs (n = 3). The symbol * indicates statistically significant differences (*P* < 0.05), **P < 0.01 and ***P < 0.001.

Our research demonstrated that the level of phosphorylation of mTOR and its downstream p70 S6K and 4E-BP1 was generally turned down by TGEV infection (**Figure 3A**). Given that mTOR acts as the regulatory center of various innate immune responses, we wonder whether mTOR could modulate TGEV replication. IPEC-J2 cells were treated with mTOR specific activator-MHY1485 and inhibitor-RAPA, followed by infection with TGEV. We observed that the inhibition of cell proliferation induced by TGEV was alleviated by MHY1485 pretreatment (**Figure 3A**), but not RAPA pretreatment (**Figure 4A**). At the same time, MHY1485 pretreatment markedly curbed the replication of TGEV (**Figure 3B**). By contrast, RAPA pretreatment promoted the viral replication at 48 hpi in IPEC-J2 cells (**Figure 4B**).

With further research, we found that the protein expression of p-mTOR and its downstream p-p70 S6K and p-4E-BP1 significantly increased with MHY1485 pretreatment (**Figures 3C, D**), while RAPA inhibited the p-mTOR, p-p70 S6K and p-4E-BP1 protein expressions regardless of the presence or absence of viral infection (**Figures 4C, D**). These data indicated that pharmacological manipulation of mTOR could control the yield of TGEV.

### mTOR Activator Augments STAT1 and ISGs Expressions in TGEV-Infected IPEC-J2 Cells

**Figure 5:**
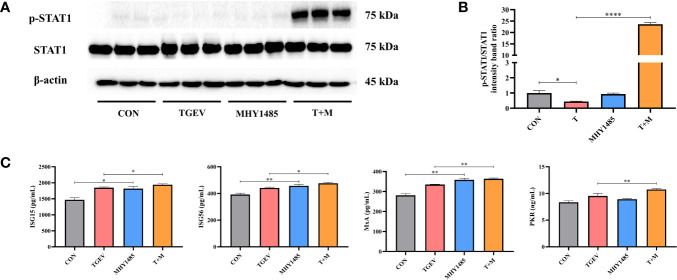
The inhibition of STAT1 and ISGs induced by TGEV infection was alleviated by MHY1485 pretreatment. **(A)** Both p-STAT1 and STAT1 levels were detected by western blot. **(B)** Quantitation of bands to demonstrate the protein level of p-STAT1/STAT1. Data express the means ± SEMs (n = 3). **(C)** The expressions of ISGs (ISG15, ISG56, MxA and PKR) were detected by ELISA. Data express the means ± SEMs (n = 3). The symbol * indicates statistically significant differences (*P* < 0.05), **P < 0.01 and ****P < 0.0001.

To investigate how mTOR modulates STAT1 activity, IPEC-J2 cells were pretreated with mTOR activator-MHY1485, followed by TGEV infection. Western blot analysis revealed that 10 μM MHY1485 significantly elevated the protein expression of p-STAT1 (**Figures 5A, B**). Similarly, the concentrations of ISGs, including ISG15, ISG56, MxA, and PKR, increased under MHY1485 pretreatment (**Figure 5C**). The evidence showed that mTOR activation could upregulate the expressions of p-STAT1 and downstream ISGs.

### mTOR Inhibitor Reduces STAT1 and ISGs Expressions in TGEV-Infected IPEC-J2 Cells

**Figure 6:**
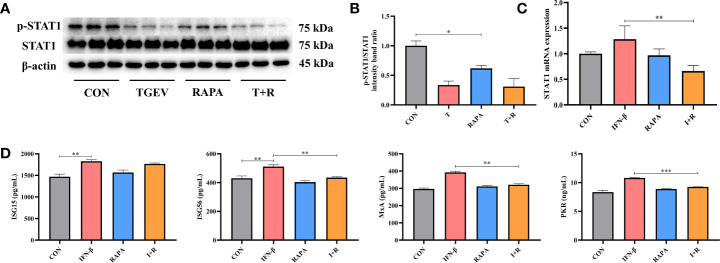
The expressions of STAT1 and ISGs was lessened by RAPA pretreatment. **(A)** Both p-STAT1 and T-STAT1 levels were detected by western blot. **(B)** Quantitation of bands to demonstrate the protein level of p-STAT1/STAT1. Data express the means ± SEMs (n = 3). **(C)** The mRNA expression of STAT1 were analyzed *via* RT-qPCR. Data express the means ± SEMs (n = 4). **(D)** The expressions of ISGs (ISG15, ISG56, MxA and PKR) were detected by ELISA. Data express the means ± SEMs (n = 3). The symbol * indicates statistically significant differences (*P* < 0.05), **P < 0.01 and ***P < 0.001.

Considering the promotion of TGEV replication triggered by mTOR inhibitor-RAPA, we clarified whether RAPA could suppress STAT1 and ISGs expressions to interrupt innate immune response against TGEV infection. It is shown that p-STAT1 level was not decreased by 10 nM RAPA pretreatment in TGEV-infected cells (**Figures 6A, B**). However, compared with the control group, 10 nM RAPA significantly inhibited p-STAT1 expression with non-infection (**Figures 6A, B**). To further confirm the effect of RAPA on STAT1 expression, 1,000 U/ml IFN-β was treated in the IPEC-J2 cells. The result showed that 10 nM RAPA markedly blocked the STAT1 activation by IFN-β (**Figure 6C**). Similarly, the concentrations of ISG56, PKR, and MxA were significantly decreased in RAPA-pretreated cells in response to IFN-β (**Figure 6D**). These observations suggested that mTOR inhibition could downregulate the expressions of p-STAT1 and downstream ISGs.

### Leu Activated mTOR Signaling and Prevents TGEV Replication

**Figure 7:**
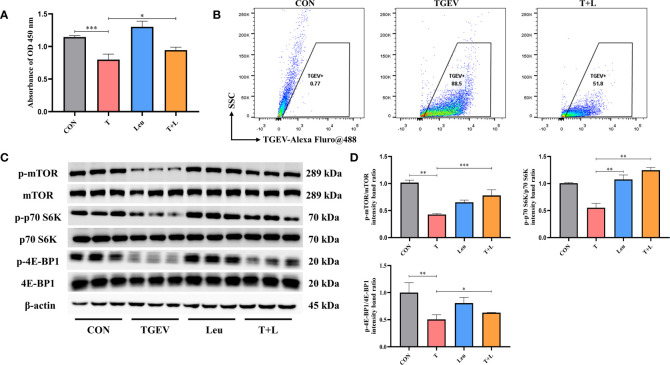
TGEV replication was regulated by Leu treatment. **(A)** CCK-8 assay was used to detect the proliferation of IPEC-J2 cells pretreated with 10 mM Leu, followed by TGEV infection. Data express the mean ± SEM (n = 3). **(B)** The contents of TGEV were analyzed *via* flow cytometry in the IPEC-J2 cells pretreated with 10 mM Leu, followed by TGEV infection. **(C)** The protein levels of p-mTOR, mTOR, p-p70 S6K, p70 S6K, p-4E-BP1 and 4E-BP-1 were detected by western blot. **(D)** Quantitation of bands to demonstrate the protein level of p-mTOR/mTOR, p-p70 S6K/p70 S6K and p-4E-BP1. Data express the means ± SEMs (n = 3). The symbol * indicates statistically significant differences (*P* < 0.05), **P < 0.01 and ***P < 0.001.

As shown in **Figure 7A**, Leu pretreatment mitigated the reduction of cell proliferation by TGEV infection. At the same time, we observed that Leu significantly reduced the contents of TGEV (**Figure 7B**) and alleviated the inhibition of p-mTOR, p-p70 S6K and p-4E-BP1 with TGEV infection (**Figures 7C, D**). Therefore, Leu may protect IPEC-J2 cells from TGEV replication by activating mTOR signaling.

### Leu Boosts STAT1 and ISGs Expressions in TGEV-Infected IPEC-J2 Cells

**Figure 8:**
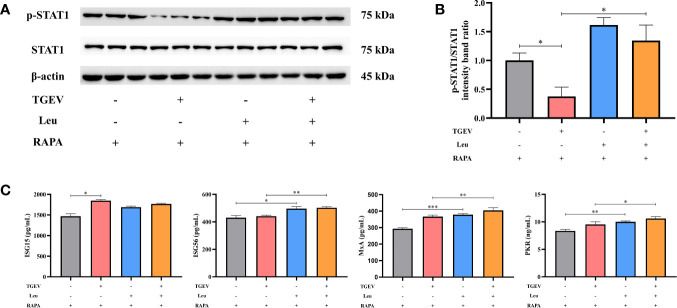
The expressions of STAT1 and ISGs increased by Leu and RAPA pretreatment in TGEV-infected cells. **(A)** Both p-STAT1 and T-STAT1 levels were detected by western blot. **(B)** Quantitation of bands to demonstrate the protein level of p-STAT1/STAT1. Data express the means ± SEMs (n = 3). **(C)** The expressions of ISGs (ISG15, ISG56, MxA and PKR) were detected by ELISA. Data express the means ± SEMs (n = 3). The symbol * indicates statistically significant differences (*P* < 0.05), **P < 0.01 and ***P < 0.001.

In this study, we investigated whether Leu could increase the STAT1 and ISGs expressions to counteract virus infection. As shown in **Figures 8A, B**, the reduction of p-STAT1 was significantly alleviated by Leu in TGEV-infected cells under the RAPA pretreatment. As expected, Leu remarkably enhanced the concentrations of ISG56, MxA, and PKR in infected IPEC-J2 cells (**Figure 8C**).

## Discussion

TGEV is known to the main etiological agent to cause diarrhea, dehydration, and high mortality of piglets, and leads to devastating economic losses in the swine industry. Once piglets are infected, the virus enters the digestive tract and destroys the small intestinal epithelial cells of piglets, affecting the absorption of nutrients (28). The construction of TGEV-infected IPEC-J2 cells, which emulates the *in vivo* intestinal environment of piglets infected by TGEV, is an ideal model for studying the mechanism of viral infection (29). In the present study, a large number of necrotic cells were observed in the culture plate, and virulence reached the highest level at 48 hpi. Moreover, the results of flow cytometry showed that the contents of TGEV increased in a time-dependent manner. Consequently, it provides a reference for the selection of appropriate time points for our subsequent experiments.

Virus infection activates the host innate and adaptive immunity to resist virus invasion. Innate immunity is the first line of defense in host protection (30). The host utilizes PRRs to detect the PAMPs of the invading virus and induces the expressions of some effector molecules through a series of signal transduction (14). ISGs play an important role in the process of direct resistance to viral infection, and JAK/STAT signaling pathway mediates the expressions of ISGs (31). STAT1, a member of the STAT family, is an important effector molecule that mediates type I IFN response (32). However, IFN-β has multi-function of antiviral infection and immunosuppression but shows less cytotoxicity. Thus, IFN-β is inclined to clinical application (33). Considering its superiority, IFN-β was selected as a positive control to activate STAT1. In order to circumvent the host innate immunity, the virus has developed a variety of strategies to inhibit the activation of antiviral effector molecules, especially to reduce the expressions of IFNs and inhibit the IFN signaling pathway (34, 35). In the present study, TGEV infection significantly inhibited the expression of STAT1 under normal conditions and the expressions of p-STAT1 and STAT1 induced by IFN-β at 48 hpi. At the same time, the expressions of ISGs, including ISG56, MxA, PKR, and OASL were also inhibited. Therefore, these results indicate that STAT1 is a vital signal transduction target against TGEV infection. TGEV invasion can block IFN signal transduction by suppressing STAT1 activity.

The mTOR signaling pathway, as the center of various important physiological processes, has been a research hotspot (36). A great deal of evidence supports the notion that the mTOR signaling pathway plays an important role in viral infection, replication, particle assembly and release (37, 38). Our study showed that the mTOR activator (MHY1485) markedly inhibited TGEV contents and mitigated the suppression of p-mTOR, p-p70 S6K and p-4E-BP1 induced by TGEV infection. Conversely, mTOR inhibitor (RAPA) promoted the TGEV infection and has an adverse impact on the expressions of p-mTOR, p-p70 S6K and p-4E-BP1 induced by TGEV infection. In addition, recent reports have revealed the points that mTOR is able to regulate STAT1 activity, suggesting the possibility of a specific cell type cascade between mTOR and STAT1 pathways to synergistically regulate the host immune response (39, 40). A recent study demonstrated that RAPA pretreatment could inhibit STAT1 nuclear translocation in primary human fetal astrocytes infected with neurotropic polyomavirus JC (JCV) (41). Therefore, we hypothesize that the resistance of TGEV infection by mTOR upregulation is mediated by STAT1 regulation. In this study, IPEC-J2 cells were pretreated with mTOR specific activator and inhibitor, respectively, to detect the STAT1 activity and the concentrations of downstream ISGs in TGEV-infected cells. It is reported that mTOR activator-MHY1485 significantly alleviated the inhibitory effect of TGEV on p-STAT1 expression, and increased the expressions of ISGs to achieve the purpose of inhibiting TGEV proliferation. On the contrary, compared with the control group, the mTOR inhibitor-RAPA inhibited the expression of p-STAT1. However, p-STAT1 level was not decreased by RAPA pretreatment in TGEV-infected cells. It is speculated that TGEV and RAPA both act as antagonists of STAT1. Under the circumstances of STAT1 suppression by TGEV infection to evade innate immunity, the negative effect of RAPA on STAT1 was not obvious in TGEV-infected cells. Moreover, RAPA significantly inhibited the expression of STAT1 in IFN-β-driven cells, consistent with the trend of expressions of ISGs determined by ELISA. This is a unique example of the positive regulation of STAT1 activity by mTOR, mechanistically highlighting the multifaceted control of TGEV replication.

BCAAs enhance the intestinal immune defense system by maintaining mucosal immune homeostasis and increasing the level of immunoregulatory cytokines (42). Leu participates in various cellular signal transduction mechanisms to regulate intestinal growth, integrity and immune function against virus invasion. Rag guanosine triphosphatases (GTPase) located on lysosomes can sense the changes in amino acids and transfer them to the mTORC1 pathway. With sufficient amino acids, mTORC1 is recruited to play a role in lysosomes (43). Our research showed that Leu could alleviate the inhibition of p-mTOR, p-p70 S6K and p-4E-BP1 and reduce the content of TGEV. The results above indicated that mTOR activation significantly increased the expression of p-STAT1 and attenuated the TGEV infection. Furthermore, we confirmed that Leu could regulate the expressions of STAT1 and ISGs by enhancing the activity of mTOR, which is coherent with the results caused by MHY1485. Overall, our findings clearly demonstrate that Leu may promote STAT1 and ISGs expressions through activating mTOR signaling, with the consequence of alleviation of TGEV infection in enterocytes.

**Figure 9:**
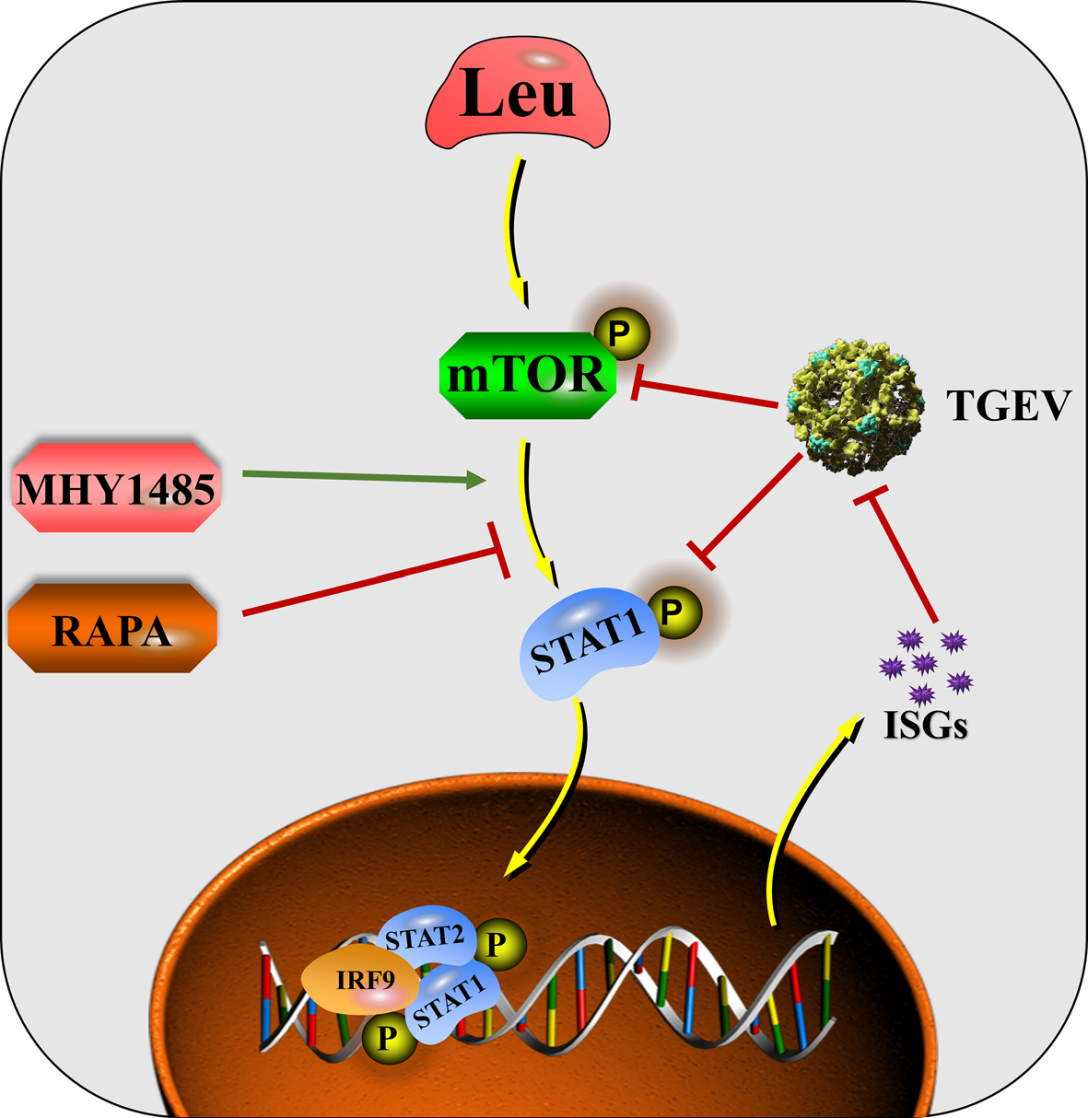
The mechanisms by which Leu affects TGEV infection through regulating mTOR signaling in IPEC-J2 cells.

In conclusion, mTOR regulation is involved in the process of innate immunity against TGEV invasion. The ability of IPEC-J2 cells to prevent TGEV infection can be altered by regulating mTOR signaling. The mechanism by which Leu alleviates TGEV infection is related to its activation of mTOR signaling and promotion of STAT1 and ISGs expressions (**Figure 9**). Harnessing an effective nutrient strategy provides a novel theoretical basis for targeting mTOR/STAT1 activation in the prevention of transmissible gastroenteritis.

## Data Availability Statement

The original contributions presented in the study are included in the article/**Supplementary Material**. Further inquiries can be directed to the corresponding author.

## Author Contributions

JD designed, performed, and analyzed the experiments and data. DC, BY, JH, JY, XM, YL, and PZ developed reagents and helped with experiments. JL acquired the funding. JD wrote the original manuscript. JL reviewed the manuscript. All authors contributed to the article and approved the submitted version.

## Funding

This work was supported by the National Natural Science Foundation of China (NSFC) (31702124).

## Conflict of Interest

The authors declare that the research was conducted in the absence of any commercial or financial relationships that could be construed as a potential conflict of interest.
