# Supplementary material for: L-Leucine Promotes STAT1 and ISGs Expression in TGEV-Infected IPEC-J2 Cells via mTOR Activation
Source: Front Immunol. 2021 Jul 22;12:656573. doi: 10.3389/fimmu.2021.656573 (PMC8339710; doi:10.3389/fimmu.2021.656573)
Supplement: Supplementary file 1 [file Table_1.docx]

TABLES

Table S1 Primers used for RT-qPCR

| Primer names | Sequence (5'→3') | | GenBank accession no. |
| --- | --- | --- | --- |
| STAT1 | F | CAGAACGGAGGCGAACCTTA | NM_213769.1 |
|  | R | AGGTTCTGGGGCTTCCTTTG |  |
| GAPDH | F | GAGTGAACGGATTTGGCCG | NM_001206359.1 |
|  | R | CACCCCATTTGATGTTGGCG |  |
